# Identification of Cyt2Ba from a New Strain of *Bacillus thuringiensis* and Its Toxicity in *Bradysia difformis*

**DOI:** 10.1007/s00284-020-02018-y

**Published:** 2020-07-03

**Authors:** Fan-Fan Wang, Shao-Xuan Qu, Jin-Sheng Lin, Hui-Ping Li, Li-Juan Hou, Ning Jiang, Xin Luo, Lin Ma

**Affiliations:** 1 Vegetable Research Institute, Jiangsu Key Laboratory for Horticultural Crop Genetic Improvement, Jiangsu Academy of Agricultural Sciences, Nanjing, 210014 China; 2 Institute of Life Science, Jiangsu University, Zhenjiang, 210023 China

## Abstract

*Bradysia difformis* is one of the most damaging pests in mushroom production in China. In this study, eight *Bacillus thuringiensis* strains were analyzed for insecticidal activity in *B. difformis*. The strain JW-1 showed the highest insecticidal activity against *B. difformis* larvae, but did not inhibit the mycelial growth of *Pleurotus ostreatus* and *P. geesteranus*. The 16S rRNA gene (1397 bp) and *cyt2* gene (792 bp) were obtained from strain JW-1. The phylogenetic tree based on 16S rRNA gene and Cyt2 toxin showed that strain JW-1 was a member of *B. thuringiensis* and Cyt2 toxin belonged to Cyt2Ba toxin cluster. The Cyt2Ba toxin from strain JW-1 was overexpressed in *E. coli* as a fusion protein and the fusion protein (70 kDa) was purified by Ni-IDA affinity chromatography. The purified Cyt2Ba fusion protein was toxic to *B. difformis* larvae (LC_50_ was 2.25 ng/mL). The identification of Cyt2Ba from strain JW-1 and confirmation of the insecticidal activity of Cyt2Ba in *B. difformis* provided a new means of biological control of the important pest in mushroom production.

## Introduction

*Bradysia difformis*, belonging to the Sciaridae family, is one of the most destructive greenhouse pests, primarily affecting edible mushroom cultivation [1]. Adult female flies lay eggs on the surface of the growing mushrooms, and the larvae mostly eat hyphae and fruiting bodies, directly damaging the mushroom produce [2]. Adult flies also spread fungal spores and mites when flying over mushrooms, which indirectly leads to lower yields [3]. *Bradysia* spp. can be controlled by traditional chemical agents because of their simplicity, effectiveness, and speed [4]. However, resistance and residual insecticide pose major threats to the mushroom industry [5, 6]. The current trend is to shift to integrated and eco-friendly pest-management, involving microorganisms with insecticidal activity, such as *Bacillus thuringiensis* [7, 8].

*Bacillus thuringiensis* (Bt) is a gram-positive, endospore-forming, ubiquitous soil bacterium that produces parasporal crystals during the sporulation phase of its growth cycle [9, 10]. These parasporal crystals contain different types of proteins with insecticidal activity, the insecticidal crystal proteins (ICP) [11]. Different ICPs possess highly specific insecticidal activities against numerous target insects, such as Lepidoptera, Diptera, Coleoptera, Hymenoptera, Homoptera, as well as some invertebrates such as Nemathelminthes and Platyhelminthes [12–14]. The insecticidal specificity of *B. thuringiensis* strains is determined by *cry* and *cyt* genes. These genes code for insecticidal crystal (Cry) protein of 65–145 kDa and cytolytic (Cyt) proteins of 25–28 kDa, which exert cytolytic activity against a broad spectrum of eukaryotic cells [15, 16]. Approximately 825 *cry* and *cyt* genes have been discovered (https://www.lifesci.sussex.ac.uk/home/Neil_Crickmore/Bt/).

Compared to Cry toxins, Cyt toxins show insecticidal activity only in Diptera and may suppress the resistance to Cry toxins [17–19]. Cyt toxins are composed of a single α–β domain and are classified into Cyt1, Cyt2, and Cyt3 [20, 21]. The size of Cyt2 (about 29 kDa) is slightly larger than that of Cyt1 (about 27 kDa) and contains an additional 15-residue sequence at the C-terminus [22]. Furthermore, Cyt2 toxin is readily expressed and forms cytoplasmic inclusions in bacteria without the need for a “helper” protein-like Cyt1 [23]. Cyt2 toxins act synergistically with Cry4Aa, Cry4Ba, and Cry11Aa [24, 25]. Proteolytically activated Cyt2Ba exhibits higher toxicity against *Anopheles*, *Culex,* and *Aedes* larvae than Cyt1Ab from *B. thuringiensis* subsp. *medellin* [26].

JW-1 is a Bt strain with insecticidal activity against *B. difformis* and contains a *cyt2* gene. At present, there are no sufficient studies on Cyt2Ba toxin against *B. difformis* on edible mushrooms. In this study, the *cyt2* gene from JW-1 was expressed in *Escherichia coli* and purified to determine its insecticidal activity on *B. difformis* under laboratory conditions.

## Materials and Methods

### *Bradysia difformis*

*Bradysia difformis* eggs were initially collected from a mushroom greenhouse in Jiangsu Province, China, in 2014. The larvae were fed mushroom mycelia and placed in plastic boxes in the laboratory. Adult flies oviposited on the surface of mycelium. The third instar larvae were used in insecticidal activity assays. All experiments were conducted at 25 ± 1 °C and relative humidity at 65 ± 5%.

### Bacterial Strains, Plasmid, and Culture Conditions

Eight Bt strains were isolated from the soil collected from the Purple Mountain of Nanjing City, Jiangsu Province, China (GPS locality: N118° 86′ 16.94″; E32° 07′ 44.11″). The strain *B. thuringiensis* var. *israelensis* (Bti) was isolated from the Bti wettable powder from Wuhan Kernel Bio-tech Co., Ltd. (Wuhan, China). For assessment of insecticidal activity, Bt strains were grown in beef extract-peptone-glucose broth (BPG medium; beef extract: 3 g/L, peptone: 5 g/L, and glucose: 10 g/L) for 72 h at 30 °C, 220 rpm. *E. coli* were cultured in Luria–Bertani (LB) broth at 37 °C, 220 rpm. The plasmids and bacterial strains used in this study are listed in Table 1.

**Table 1:** Bacterial strains and plasmids

Strain or plasmid	Relevant characteristics	Source
Bt strains		
JW-1, 51-4, 68-6, 69-1, 69-3, 88-1, 99-3, 214-4	Wild-type strains with insecticidal activity	This study
Bti	Purified from *Bacillus thuringiensis* subsp. *israelensis *wettable powder	Wuhan Kernel
Edible fungus strains	*Pleurotus ostreatus*, *Pleurotus geesteranus*, *Auricularia polytricha*	This study
*Escherichia coli*		
TOP10	F- *mcrA* Δ (*mrr*-hsd RMS-*mcr*BC) *φ*80 *lac*ZΔM15 △l*ac*X74 *rec*A1 *ara*Δ139Δ(*ara-leu*)7697 *gal*U *gal*K rps (Strr) endA1 *nup*G	Zoonbio
Arctic	F–*ompT hsdS*(rB– mB–) *dcm* + Tetr *gal λ*(DE3) end A Hte [cpn10 cpn60 Gentr] [argU proL Strr]	
Plasmids		
PMAL-c5x	Expression vector for *cyt2*, MBP, Ap^r^	Zoonbio
pCYT-2	pMAL-c5x carrying 792-bp PCR product of the *cyt2*, Ap^r^	This study

### Insecticidal Activity Assay

Insecticidal toxicity of the eight Bt strains and their Cyt2 toxins were analyzed in third instar larvae of *B. difformis* by larval feeding assay [27]. Single colonies of the eight Bt strains were individually inoculated into 5 mL of BPG broth overnight at 30 °C, 200 rpm, and 0.5 mL of this culture solution was then transferred to 50 mL of BPG broth and inoculated again for 72 h using the same culture conditions. The liquid cultures of the strains were normalized to 0.7 at OD_600_ using the BPG broth. Three fresh pieces (diameter of 1 cm) of fruiting *A*. *polytricha*, soaked in Bt strain normalized broth (2 mL) or Cyt2 toxin diluent (2 mL) were placed on a 9-cm plate. One piece of the filter paper (Sangon Biotech, Shanghai, China), wetted by 0.8 mL of same liquid as used for *A. polytricha*, was laid on the bottom of the plate. Thirty larvae were transferred into every plate and kept at 25 ± 1 °C and 65 ± 5% relative humidity for 72 h. All assays were performed in triplicates. BPG broth was used as a control, and the Bti strain was used as a positive control. Mortality and correct mortality were calculated [27], and statistical analysis was performed [28].

### Antifungal Activity Assay

The effects of Bt strains that showed the highest insecticidal activity against *B. difformis* on the hyphal growth of *Pleurotus ostreatus* and *P. geesteranus* were determined by the confrontation culture method [29]. A PDA disc (5 mm in diameter) containing the mycelia above was placed onto the center of each 9-cm plate. A 5-μL aliquot of Bt strain fermentation broth was immediately spotted onto the same plate equidistant from the edge of the fungal disc. Bti fermentation broth was used as a control. Three replicates of plate bioassays were performed independently. The inoculated plates were incubated in the dark for one week at 25 ± 1 °C.

### 16S rRNA Gene and *cyt2* Gene Analysis

The single colony of Bt strain JW-1 was incubated in 5 mL of LB broth overnight at 30 °C, 200 rpm. The bacterial cells were resuspended in 600 μL sorbitol buffer (1.2 M with 0.1 M phosphate-buffered saline (PBS) and 5 μL lysozyme (0.05 g/mL) and kept at 30 °C overnight in the water bath. Total· DNA of JW-1 was extracted using the Bacterial Genome Extraction Kit (TsingKe, Nanjing, China).

The 16S rRNA gene of the strain JW-1was amplified using the universal primers 27F and 1492R [30]. According to the complete *cyt2Ba* gene of strain HD-567 (GenBank accession no. GQ919039.1), primers JW-1-cyt2F/R (JW-1-cyt2F: 5′-CCATATGGATGCACCTTAATAATTTGAA-3′; JW-1-cyt2R: 5′-CCCGCTTGTTACGATTTTATTGGATTAACAT-3′) were designed using the Primer Premier 5 software [31] and were used to amplify the full *cyt2* gene of JW-1. Amplification was carried out with I-5™ Hi-Fi DNA Polymerase (TsingKe Company). The cycling program for PCR of the 16S rRNA gene included 35 cycles of 98 °C for 10 s, 50 °C for 10 s, and 72 °C for 15 s and the cycling program for PCR of the *cyt2* gene included 35 cycles of 98 °C for 10 s, 50 °C for 10 s, and 72 °C for 10 s. Sequencing reads of 16S rRNA and *cyt2* were assembled by DNASTAR Lasergene package version 7.1.0, and the DNA nucleotide sequences of 16S rRNA and *cyt2* were deposited in the GenBank database under the Accession Numbers (MN539648.1 and MN539642.1). Phylogenetic trees were constructed based on 16S rRNA gene and Cyt2 toxin sequences of JW-1 using MEGA 6.0 software [32]. The evolutionary history was inferred by using the Maximum Likelihood method and a bootstrap analysis of 1000 replications was performed [33].

### Expression Vector Construction

To investigate the insecticidal activity of the Cyt2 toxin from JW-1, the Cyt2 protein was expressed in *E. coli*, by inserting the blunt-end PCR product (complete sequence of the 792 bp *cyt2* gene) into the multiple cloning site of pMAL-c5X, following manufacturer’s instructions, to produce the plasmid pCYT-2. pCYT-2 was then transformed into *E. coli* TOP10 competent cells, and the transformants were identified on LB agar containing ampicillin (100 μg/mL). The *cyt2* gene was amplified from the *E. coli* colonies with the primers JW-1-cyt2F/R to confirm the presence of the plasmid.

The expression of Cyt2 in *E. coli* with pCYT-2 was induced by addition of 0.5 mM of isopropyl-β-d-1-thiogalactopyranoside (IPTG) to the culture and incubation at 20 °C overnight, as described previously [34]. Following this, 1 mL of the induced bacterial solution was centrifuged at 10,000 rpm for 2 min, and the cell pellets were suspended in 100 μL of × 1 loading buffer (SDS: 20 mg/mL, bromophenol blue: 0.1 mg/mL, 1 M Tris–HCl, pH6.8: 25 μL/mL, glycerinum: 0.1 mL/mL, and adding 5 μL/mL of β-mercaptoethanol before using). The solution was centrifuged at 4000 rpm for 10 min, and the cell pellets were resuspended in PBS. After disrupting the suspension by ultrasonication, the proteins in the supernatant and the precipitate were analyzed by sodium dodecyl sulfate–polyacrylamide gel electrophoresis (SDS-PAGE) [35].

### Purification and Western Blot Analysis of Cyt2 Fusion Protein

The supernatant from the Cyt2 preparation from above was loaded onto a Ni-IDA Sepharose CL-6B affinity chromatography column pre-equilibrated with Ni-IDA binding buffer at 0.5 mL/min. The column was washed by Ni-IDA binding buffer at 0.5 mL/min and Ni-IDA washing buffer (20 mM Tris–HCl, pH 8.0, containing 20 mM imidazole and 150 mM NaCl) at 1 mL/min until the OD_280_ value of the effluent reached the baseline. The column was then eluted with Ni-IDA elution buffer (20 mM Tris–HCl, pH 8.0, containing 250 mM imidazole and 150 mM NaCl) at 1 mL/min to obtain the target protein. The collected protein solution was dialyzed overnight against tris-buffered saline (TBS) (20 mM Tris–HCl, 150 mM NaCl, pH 8.0). The dialysate was analyzed by 12% SDS-PAGE.

Western blot analysis was performed as previously described [36]. The purified recombinant Cyt2 toxin was separated by SDS-PAGE and transferred to polyvinylidene difluoride (PVDF) membrane, and then the PVDF membrane was washed four times by PBS-Tween 20 (PBST, 0.1% Tween 20) buffer and incubated with His-tag primary antibody (Zoonbio Biotechnology, Nanjing, China) diluted by 5% skim milk blocking buffer (1:1000) at 4 °C overnight. After washing three times with PBST buffer, the membrane was incubated with goat anti-mouse IgG-conjugated alkaline phosphatase (Zoonbio Biotechnology, Nanjing, China) prepared in 5% skim milk blocking buffer (1:5000) at 37 °C for 1 h. The membrane was then washed four times with PBST buffer. Images were developed by enhanced chemiluminescence (ECL). Protein concentrations were determined by Bradford protein assay (Bio-Rad Laboratories, Hercules, CA) on samples solubilized as previously described.

## Results

### Determination of Toxicity and Inhibitory Activity

**Fig. 1:**
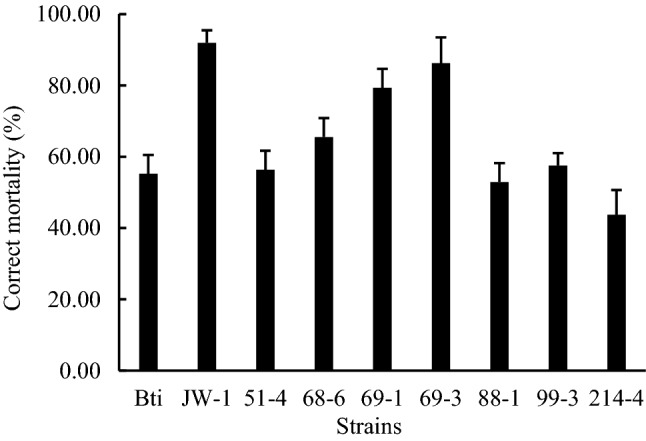
The toxicity of Bt strains in *B. difformis* larvae. Insecticidal toxicity of the eight Bt strains and their Cyt2 toxins were analyzed in third instar larvae of *B. difformis* by larval feeding assay. Bti strain was used as a positive control

Eight laboratory Bt strains that produced parasporal crystals were used to determine the virulence on the larvae (Fig. 1). After 72 h, the corrected mortality of *B. difformis* larvae treated with all bacterial strains was above 50% except for strain 214-4. Strains JW-1 and 69-3 showed the highest insecticidal activity, and the corrected mortality was 91.96 ± 3.49% and 86.21 ± 7.26%, respectively. After treatment with Bt for 72 h, most larvae became darkened or started to disintegrate.

**Fig. 2:**
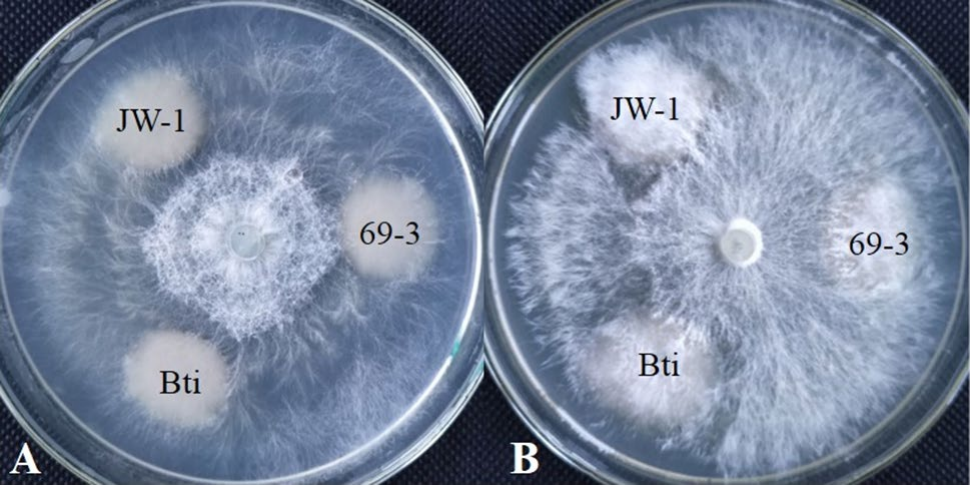
Antifungal activities of Bt strains against hyphae of *P. ostreatus* (**a**) and *P. geesteranus* (**b**). The three kinds of Bt strains (5 μl aliquot) was inoculated onto the PDA plate simultaneously with *P. ostreatus* (**a**) and *P. geesteranus* (**b**) (5 mm PDA discs with mycelia), then incubated in the dark for one week at 25 ± 1˚C

Strains JW-1 and 69–3 were tested for their antifungal activity against hyphae of *P. ostreatus* and *P. geesteranus*. The hyphae of the two fungi grew normally on the plates without showing inhibition by the bacteria. Therefore, we identified that the three Bt strains (JW-1, 69-3, Bti) did not inhibit mycelial growth (Fig. 2). Based on the results from the insecticidal and antifungal activity assays, the strain JW-1 was selected as the candidate strain for further research.

### Amplification of 16s rRNA and *cyt2* genes

**Fig. 3:**
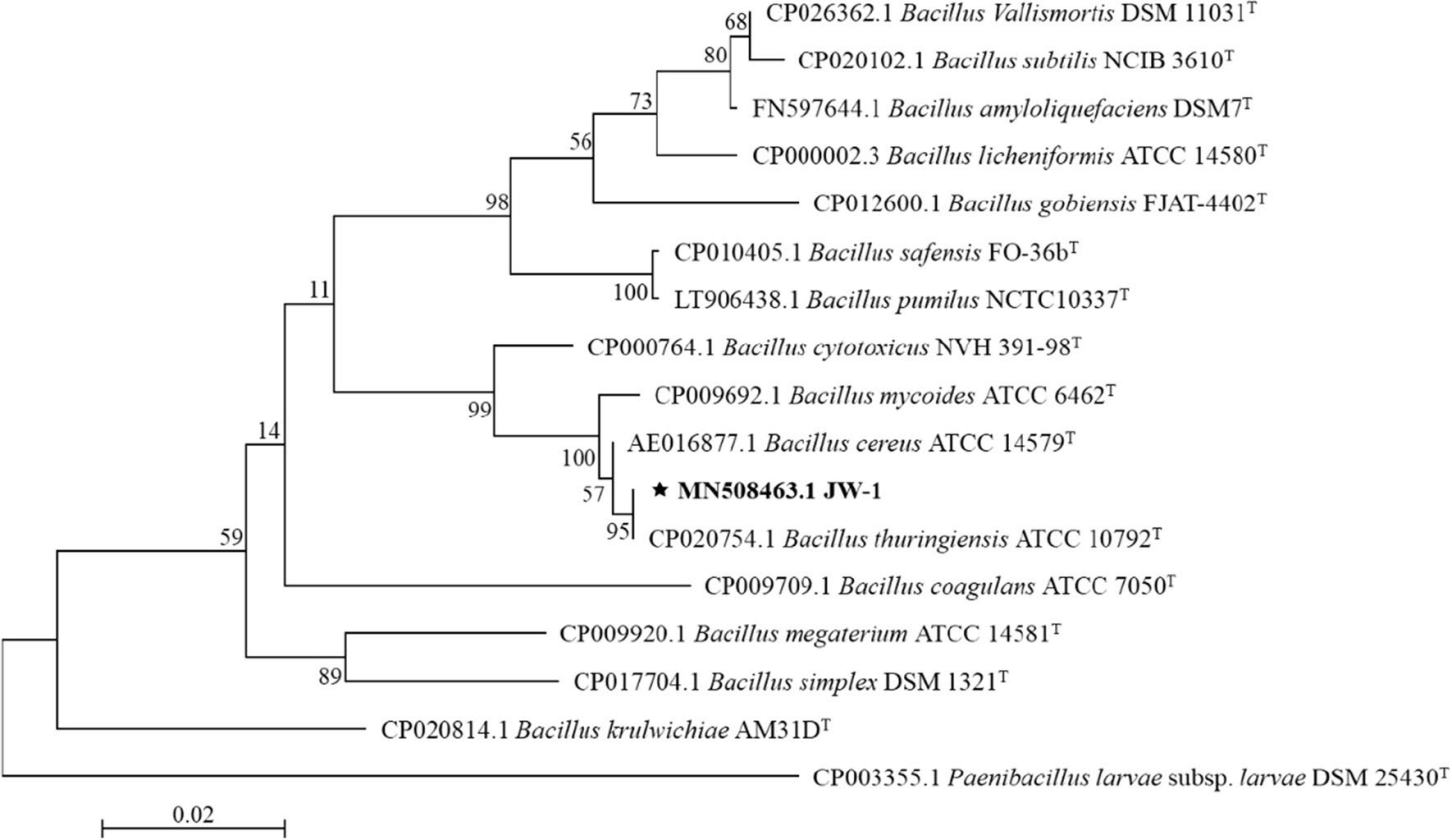
Phylogenetic tree based on 16S rRNA gene sequences. The dendrograms were generated using the maximum likelihood method and gene distances were calculated using the Jukes–Cantor model

The nearly full-length 16S rRNA gene sequence (1397 bp) of strain JW-1 shared highest homologous to *Bacillus* species archived in GenBank. The phylogenetic tree based on 16S rRNA gene showed that strain JW-1 was clustered together with *B. thuringiensis* (Fig. 3).

**Fig. 4:**
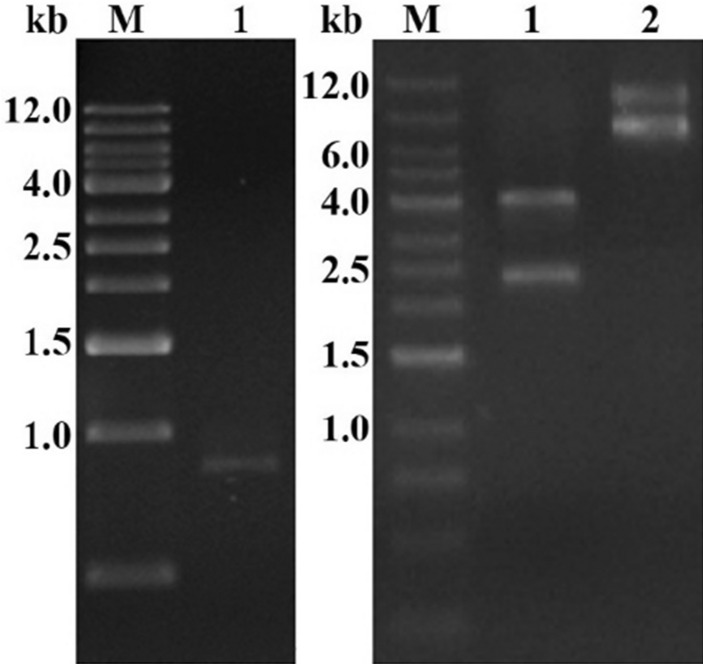
The electrophoresis result of *cyt2* and the verification of pCYT-2. **a** PCR confirmation of full-length *cyt2* gene around 0.8 kb in the JW-1 (lane 1), the primers JW-2F and JW-2R were used to amplify the *cyt2* gene. **b** pCYT-2 plasmid was digested with *PvuI* around 2.4 kb and 4.0 kb (lane 1), the pCYT-2 plasmid around 6.4 kb (lane 2)

**Fig. 5:**
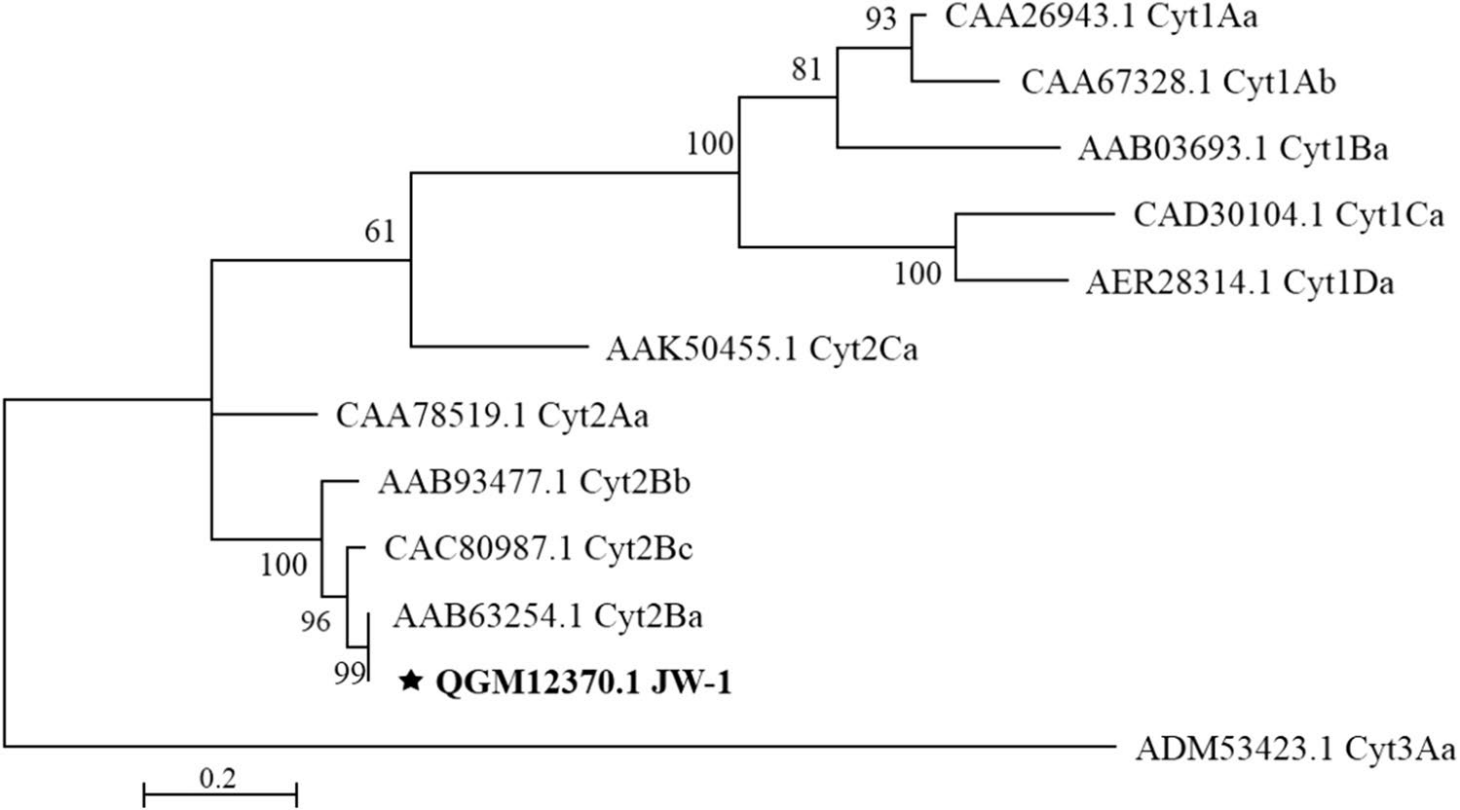
Phylogenetic tree based on Cyt2 toxin sequences. The gene distances were calculated using the Jones–Taylor–Thornton (JTT) model and dendrograms were generated using the maximum likelihood method. Initial tree for the heuristic search were obtained automatically by applying Neighbor Joining

A 792 bp fragment was obtained from PCR amplification of the *cyt2* gene from the total DNA of Bt strain JW-1 (Fig. 4a). The Cyt2 toxin sequence (GenBank accession no. QGM12370.1) exhibited 99% identity to the reported Cyt2Ba toxin, confirming that this protein is a member of Cyt2Ba in the Cyt2 family (Fig. 5).

### Construction of Expression Vector

The entire *cyt2Ba* (792 bp) gene was inserted into pMAL-c5x by seamless cloning. *PvuI* was used for double enzyme digestion, confirming the full-length of pCYT-2 construct. The result of digestion was 2.4 kb (0.8 kb *cyt2Ba* and 1.6 kb located at vector) and 4.0 kb vector (Fig. 4b).

### Induced Expression

**Fig. 6:**
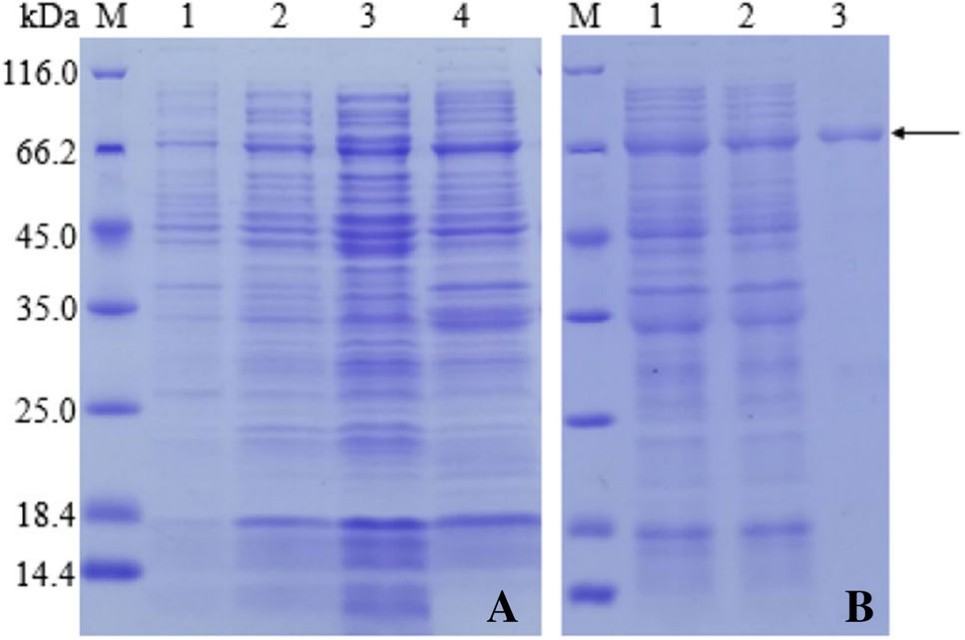
SDS-PAGE analysis of expression (**a**) and fusion protein purification (**b**). **a** Protein expression before induction (lane 1), protein expression after induction (lane 2), the supernatant of induced fragmentation (lane 3), precipitation of induced fragmentation (lane 4). **b** Sample of pulverization (lane 1), effluent sample (lane 2), elution sample (lane 3)

*Escherichia coli* Arctic culture (pCYT-2) was incubated with IPTG in LB medium. The toxin Cyt2Ba (30 kDa) was expressed as a fusion protein with a 40 kDa maltose-binding protein (MBP) tag. The 70 kDa recombinant product was visualized in SDS-PAGE, which validated the expression of Cyt2Ba toxin (Fig. 6a). Due to the protein structure, the protein appeared to be smaller than the calculated molecular weight. The results of SDS-PAGE indicated that Cyt2 was mainly present in the supernatant and expressed in soluble form, which could be directly purified by affinity chromatography.

### Purification of the Fusion Protein and Western Blot Verification

**Fig. 7:**
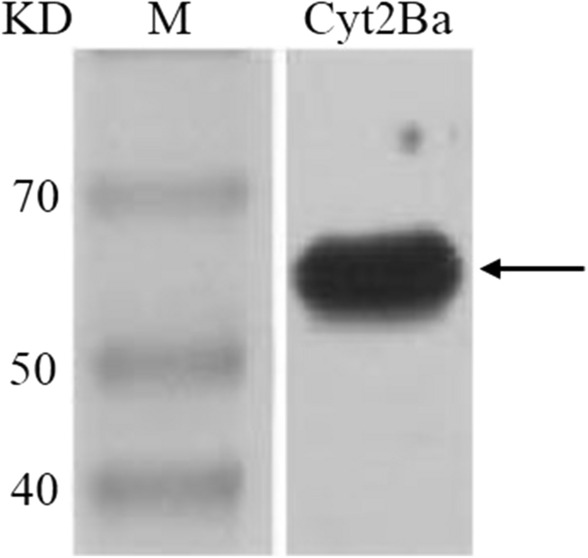
Western Blot analysis of fusion protein purification

After the fusion protein was purified by Ni column affinity chromatography, the initial bacterial homogenate, effluent, and eluate were separately subjected to 12% SDS-PAGE analysis. The result revealed that a relatively pure target protein fraction was obtained in the eluted fractions (Fig. 6b). After the purified protein was subjected to Western blotting (Fig. 7), the fusion protein was solubilized on the Western blot.

### Insecticidal Activity Assay of Cyt2Ba Toxin

Bioassays were performed against the third instar larvae of *B. difformis*. After 72 h of treatment, the mortality caused by different doses of Cyt2Ba toxin showed a linear relationship between the log dose and probe mortality. The regression equation of Cyt2Ba toxin was *y* = 4.49 + 1.45*x* (*x* means the logarithm value of concentration; *y* means the probability value of mortality). The LC_50_ value was 2.25 ng/mL, where 95% fiducial limit for LC_50_ was between 1.44–3.50 ng/mL and the *R* value was 0.9433.

## Discussion

In China, the mushroom cultivation scales range from technologically assisted industrialized production to conventional field production. Because of the short reproduction cycle and the high resistance developed to pesticides, *B. difformis* damages are more severe in simple mushroom cultivation settings. Heat stress and some biological control microorganisms, such as *Beauveria bassiana* and entomopathogenic nematodes (EPNs), showed the control efficacies against *B. difformis* in soil or spawned mushroom compost [37–39]. However, these methods are not effective for controlling *B. difformis* in simple mushroom cultivation in China, because of the difficulties in adequate control of temperature, potting media, and application dose [40]. In contrary Bt strains have already been used in production of mushrooms to control *B. difformis* and are readily accessible to growers due to its broad applicability.

Bt is an effective agent to control mosquitoes due to production of multiple toxins with different modes of action [41]. Cyt toxins in Bt synergize with the toxic effect of Cry toxins. Cyt toxins have insecticidal activity and function as a receptor to synergize the Cry11 toxicity [42, 43]. Besides, at least two Cyt toxins are present in the Bt strains to control the Dipteran insects, such as *B. thuringiensis* subsp. *israelensis* and subsp. *morrisoni* [19].

In this study, the strain JW-1, as a member of *B. thuringiensis*, showed the obvious insecticidal activity against *B. difformis* larvae and did not affect the hyphal growth of mushroom. Cyt2Ba, composed of 263 amino acids, is a minor component within the complex of the crystal proteins expressed in the Bt strain [44]. The toxicity of the recombinant Cyt2Ba toxin from strain JW-1 in *B. difformis* larvae was confirmed by biological assay. The larvicidal activity of recombinant Bt toxin Cyt2Ba against mosquito larvae, such as *Aedes albopictus* and *Culex pipiens quinquefasciatus*, has been reported [45], but there are few research for mushroom pest control. The results from this study showed that *B. difformis* damage in mushroom cultivation could be controlled by this Bt toxin as an alternative to minimize the use of chemical insecticides. Further work on the biological activity of Cyt2 toxin and its combination with other Cyt and Cry toxins will help in our understanding of the mechanisms. This knowledge may have a significant impact on biological insect resistance-management programs.
